# Post-traumatic stress disorder in children and adolescents one year after a super-cyclone in Orissa, India: exploring cross-cultural validity and vulnerability factors

**DOI:** 10.1186/1471-244X-7-8

**Published:** 2007-02-14

**Authors:** Nilamadhab Kar, Prasanta K Mohapatra, Kailash C Nayak, Pratiti Pattanaik, Sarada P Swain, Harish C Kar

**Affiliations:** 1 Wolverhampton City Primary Care Trust, Corner House Resource Centre, 300, Dunstall Road, Wolverhampton, WV6 0NZ, UK; 2 Mental Health Institute, SCB Medical College, Cuttack, India; 3 Department of Psychiatry, VSS Medical College, Burla, India; 4 Department of Clinical Psychology, Mental Health Institute, SCB Medical College, Cuttack, India; 5 SVP Postgraduate Institute of Paediatrics, Cuttack, India; 6 Directorate of Statistics and Economics, Government of Orissa; Bhubaneswar, India; 7 Quality of Life Research and Development Foundation, Bhubaneswar, India

## Abstract

**Background:**

It has been asserted that psychological responses to disasters in children and adolescents vary widely across cultures, but this has rarely been investigated. The objectives of the study were to clinically evaluate the construct of traumatic stress symptoms and disorder in children and adolescents after a super-cyclone in Orissa, India; to find out the prevalence at one year; compare the effect in high and low exposure areas and study the factors associated with it.

**Methods:**

Clinical examination of children and adolescents (n = 447) was done, supplemented by a symptoms checklist based on International Classification of Mental and Behavioural Disorders, Diagnostic Criteria for Research and a semi-structured questionnaire for disaster related experiences.

**Results:**

A majority of children had post-traumatic symptoms. Post-traumatic stress disorder (PTSD) was present in 30.6% (95% confidence interval: 26.4 to 34.9), and an additional 13.6% had sub-syndromal PTSD. Parents or teachers reported mental health concerns in 7.2% subjects, who were a minor proportion (12.8%) of subjects with any syndromal diagnosis (n = 196). Significantly more (43.7%) children in high exposure areas had PTSD than that (11.2%) in low exposure areas (p < 0.001). Depression was significantly associated with PTSD. Binary logistic regression analysis indicated that high exposure, lower educational level and middle socioeconomic status significantly predicted the outcome of PTSD. Extreme fear and perceived threat to life during the disaster, death in family, damage to home, or staying in shelters were not significantly associated with PTSD.

**Conclusion:**

Following natural disaster PTSD is a valid clinical construct in children and adolescents in Indian set up; and though highly prevalent it may be missed without clinical screening. Its manifestation and associated factors resembled those in other cultures.

## Background

Studies have noted that post-traumatic stress disorder (PTSD) in children remains usually under-recognized; and parents, teachers and even mental health professionals significantly underestimate both the intensity and the duration of the stress reactions in children [1]. However children exposed to a high magnitude natural disaster report sufficient symptoms to establish PTSD syndrome based on diagnostic classification [2]. Current literature suggests that there are wide variations of prevalence figures for PTSD in children and adolescents after natural disasters.

Studies conducted within one year of disasters report prevalence figures for PTSD such as: 4.5% three months after the 1999 earthquake in Ano Liosia, Greece [3]; 5% three months following hurricane Hugo [2] and 3% of males and 9% females 6 months after Hurricane Andrew [4].

Studies reporting prevalence at one year following natural disaster range from no syndrome after a flood [5], to 3.8–6.2% after Hurricane Hugo [6]; 26.9% after super-cyclone of Orissa [7]; and 28.6% mild to moderate PTSD following Northridge Earthquake [8].

Similarly studies more than one year following disasters also suggest variable prevalence figures like 70% with moderate to severe post-traumatic stress symptomatology at 21 months after Hurricane Andrew [9]; 37% probable PTSD two years after Buffalo Creek dam collapse [10]; and 1.8% very severe, 20.4% severe, 38.3% moderate and 30.2% mild symptoms of PTSD and 22.2% probable PTSD three and a half years after the Marmara earthquake in Turkey [11]. These studies suggest that the symptoms of PTSD continue for years after natural disasters.

The variation in prevalence figures could be due to severity of exposure, assessment duration from the event and the methodology used [12]. All children exposed to disasters do not develop PTSD; some appear to have greater risk [2]. Various factors have been found to be predictive of development of PTSD e.g. severity of trauma exposure [13], female gender [14], trauma related parental distress [15] and accumulation of multiple stressors [16]. Previous traumatic events have been found to be a vulnerability factor for development of PTSD once they reach a certain threshold of trauma load [17,18]. Past experience of stressful events and their outcome, successful or otherwise, and coexisting adverse circumstances may also predict [19].

Manifestation of post-traumatic stress symptoms and syndrome may depend upon variation in cultural and societal response to stress, coping strategies and available support. It has been emphasized that there are numerous cultural considerations which are to be responded to in understanding and treating PTSD across cultures [20]. It has been observed that psychiatric morbidity among children and adult survivors of massive disasters in the Third World far exceed those found for disasters in United States communities [21]. However PTSD itself has been criticized as having limited cross-cultural validity [22]. PTSD as a valid clinical construct in adult survivors of natural disasters in Indian context has been suggested [23]. As one can not generalize the adult findings to children [12,24], it becomes imperative to study prevalence of and associated risk factors for PTSD in children and adolescents after disasters in different socio-cultural settings. In addition, such evaluation is important not only for the assessment of mental health, but for intervention and prevention issues as well, especially as trauma in childhood and adolescence is suggested to bring in prolonged vulnerability [25].

On the above background, we intended to find out the prevalence of post-traumatic stress symptoms and disorder, associated other symptoms and trauma related risk factors in children and adolescents following the 1999 super-cyclone in Orissa, India. We tried to find out the difference in prevalence in high and low exposure areas. The focus was also to clinically examine the construct of PTSD in children and adolescents following disasters in this culture.

### The super-cyclone of Orissa

A super-cyclone hit 12 coastal districts of Orissa, on the East Coast of India, on October 29, 1999, with a wind speed of 260 to 300 kilometer per hour and it continued for 72 hours. Tidal waves from sea at a height of 7 meters came up to 15 kilometers inland at various places. Thousands of villages were marooned for over two weeks before relief services could gain access. The super-cyclone affected over 15 million people, around 10000 persons died. The Government of Orissa estimated total damage at 39680 million Indian Rupees (around one billion US dollar) [26,27].

## Methods

The study was undertaken in the most affected Jagatsinghpur district and two other districts (Bhadrak and Kendrapara) out of 12 affected. The sea-side areas of Jagatsinghpur which were submerged in the seawater and experienced maximum impact of the cyclone with heavy loss of life and property were considered to be high exposure areas (HEA). Other districts which were hit by the super-cyclone but did not get swept away by sea water were considered low exposure areas (LEA).

Following stratification to HEA and LEA, the subjects were selected in clusters. In Jagatsinghpur district four schools from the most affected Erasama block were chosen. In the other districts one block per district was selected. The study was carried out on two schools of Kendrapara and one school in Bhadrak District. The classes in schools were selected randomly. All students in the selected classes were taken up as subjects for the study. Efforts were made to contact the children and adolescents who were absent in the class, for enrolment to the study. Some evaluations were done in their homes in villages. The sample was thus ensured to be representative of the children and adolescents of the two strata. The teachers were briefed about the study beforehand. The children were interviewed in their schools and villages in the months of November and December 2000, one year after the super-cyclone.

Informed consent from a parent or a responsible adult was obtained. There were no refusals to participate. The class teacher or a parent of the child was present during the interview. The clinical evaluation was conducted by the psychiatrists in the local language, Oriya. It was supplemented by a checklist questionnaire based on International Classification of Mental and Behavioural Disorders, 10^th ^revision: Diagnostic Criteria for Research (ICD-10-DCR) [28]. The questionnaire included the symptoms of PTSD and provided the translated version in the local language Oriya. In addition to PTSD symptomatology, we specifically assessed for depressive, anxiety and somatic symptoms using a semi-structured format.

Socio-demographic variables and disaster related experiences e.g. damage to house, displacement and living in shelters, death in family and extreme degree of fear during disaster with perceived threat to life were noted. Socioeconomic status (SES) was determined based on per capita family income per month, which was supplemented by the assessment of household income, employment status, type of housing, profile of the land owned, indebtedness and financial capability to buy food. The SES groups were categorized as 'below poverty line', lower, lower middle, upper middle and upper. Parents and teachers were asked to report any mental health concern with onset following super-cyclone in any child or adolescent. The study protocol was approved by the ethics committee of Quality of Life Research and Development Foundation.

The statistical tests were done by SPSS package. The categorical data were analyzed by using chi-square tests and the continuous variables were compared by two-tailed t-tests. Binary logistic analysis was used to determine the impact of independent variables on the diagnosis of PTSD as outcome. Statistical significance was defined at the standard 0.05 level.

## Results

### Sample

The sample consisted of 447 children and adolescents with an age range of 7–17 years (mean age ± SD: 12.9 ± 1.83 years). Sample characteristics are presented in table 1. There were none in the upper SES group. We combined lower and upper middle groups to a single middle SES group for comparison purposes. Amongst the subjects, 268 children and adolescents (female: 136 and male: 132) were from the HEA, who experienced the sea tide sweeping inland, in addition to being in the centre of the cyclone. Others (n = 179, female: 88 and male: 91) were from LEA. Mean age of the children from high and low exposure areas were 12.6 ± 1.85 and 13.6 ± 1.62 years respectively.

**Table 1:** Sample characteristics and prevalence of PTSD in different groups

Variables	Total (N = 447)		Proportion with PTSD (N = 137)		95% CI
	n	%	n	%	

Gender					
Female	224	50.1	72	32.1	25.9–38.2
Male	223	49.9	65	29.1	23.1–35.1
Age categories in years*					
10 or less	53	11.9	13	24.5	18.6–30.5
11–13	175	39.1	75	42.9	35.6–50.2
14 or above	219	48.9	49	22.4	19.6–25.2
Educational level*					
Class 5 or less	120	26.8	50	41.7	32.9–50.5
Class 6–8	110	24.6	41	37.3	28.3–46.3
Class 9–10	217	48.5	46	21.2	15.8–26.6
Socioeconomic status*					
Below poverty line	272	60.8	66	24.3	19.2–29.4
Lower	96	21.5	29	30.2	21.2–39.4
Middle	79	17.7	42	53.2	42.2–64.2
Exposure*					
High	268	59.9	117	43.7	37.8–49.6
Low	179	40.0	20	11.2	6.6–15.8
Traumatic experience					
Death in family	17	3.8	6	35.3	12.6–58.0
Had disaster warning**	186	41.6	67	36.0	29.1–42.9
Home damaged	379	84.8	116	30.6	25.9–35.2
Had to stay away from home	348	77.8	108	31.0	26.2–35.9
Had extreme degree of fear during cyclone	388	86.8	122	31.4	29.1–33.7

*p < 0.001; **p < 0.05; CI: Confidence Interval

### Cyclone related experience

Less than half of the children and adolescents studied said that they had prior information regarding the arrival of the super-cyclone (HEA 44.0% vs. LEA 38.0%). Significantly more number of subjects from HEA reported complete damage to home (89.9% vs. 77.1%, χ^2^:13.7, p: 0.000), displacement due to super-cyclone and having to stay in shelters (83.6% vs. 69.3%, χ^2^:12.7, p: 0.000), extreme degree of fear during the disaster with life threat (91.8% vs. 79.3%, χ^2^:14.5, p: 0.000) and death in family in super-cyclone (6.3% vs. 0.0%, χ^2^: 11.8, p: 0.001) compared to subjects in LEA suggesting the experience in HEA to be more traumatic.

### Prevalence of PTSD

After one year of the disaster, PTSD was diagnosed in 137 (30.6%; 95% Confidence Interval (CI): 26.4–34.9) children and adolescents. It was observed that an additional 61 (13.6%) subjects could be categorised as sub-syndromal PTSD. They had number of symptoms needed to fulfil the criteria for PTSD, but the symptoms were occasional, fleeting, lacked persistence and severity to justify syndromal PTSD diagnosis clinically. Proportions of subjects with PTSD in different socio-demographic variables and associated factors are given in table 1. Mean age of subjects with PTSD was 12.6 ± 1.69 compared to 13.2 ± 1.86 years of others (t: 2.94, p: 0.003). More children from age category of 11–13 years had PTSD. Children up to the class 5 were more vulnerable (mean age 10.5 years) than those in higher classes. Higher exposure and middle socioeconomic status were significantly associated with PTSD. Prevalence figures of various post-traumatic stress symptoms are presented in table 2, with the difference between the high and low exposure areas.

**Table 2:** Post-traumatic stress symptoms

Post-traumatic stress symptoms	Total	Exposure	
		Low (n = 179)	High (n = 268)

	%	%	%

Experiencing distress when reminded or exposed to cues	83.4	76.0	88.4**
Actual or preferred avoidance	60.0	69.3	53.7**
Distressing persistent recollection	53.0	16.8	77.2*
Difficulty recollecting some aspects of the cyclone	41.6	54.2	37.2*
Hypervigilance	41.2	7.3	63.8*
Difficulty in concentrating	36.9	15.1	51.5*
Exaggerated startle	36.2	5.6	56.7*
Irritability or outbursts of anger	24.8	1.1	40.7*
Reexperiencing, reliving	24.4	7.8	35.4*
Recurring dreams	18.8	15.6	20.9
Difficulty in falling or staying in sleep	8.3	3.9	11.2**

*p < 0.001; **p < 0.01

Parents and teachers reported mental health concerns in 32 (7.2%) subjects, amongst whom 17 (53.1%) had PTSD (χ^2^: 8.2, p: 0.004). Diagnostic breakdown of these 32 children and adolescents were: no syndromal diagnosis in 7 (21.9%), only depression in 8 (25.0%), only PTSD in 9 (28.1%) and both diagnoses in 8 (25.0%). The ones without syndromal diagnosis had post-traumatic stress symptoms and other associated symptoms but those did not amount to syndromal clinical diagnosis. While 25 (78.1%) children and adolescents out of 32 reported by parents had syndromal diagnosis, they were only 12.8% of the 196 subjects who had any diagnosis.

### Other symptoms

There are various other symptoms which were significantly associated with subjects having PTSD (table 3). Suicidal ideas were there in 4.9% and ideas of worthlessness in 6.7%. There were 106 (23.7%) subjects with syndromal depression based on ICD-10-DCR [28]. As a comorbid condition depression was found in 47 (34.3%) children and adolescents with PTSD, compared to 59 (19.0%) subjects without PTSD (χ^2^:12.3, p: 0.000). This highlighted that most of the children with PTSD did not have comorbid depression; and more than half (55.7%) of depressed children did not have PTSD. There were 196 (43.8%) children and adolescents who had any syndromal diagnosis, either PTSD or depression or both. There was no disruptive behaviour, conduct problem or oppositional behaviour noted or reported by the parents or teachers.

**Table 3:** Other symptoms significantly associated with PTSD

Symptoms	Total no of children reporting this problem		Proportion in subjects with PTSD	
	n	%	n	%

Continuing fear that cyclone may come again	204	46.3	107	52.5*
Anhedonia	142	31.8	75	52.8*
Depressed mood	126	28.2	55	43.7*
Tired most of the time	121	27.1	52	42.9***
Hopelessness	120	26.8	56	46.7*
Headache	107	23.9	32	29.9*
Decreased appetite	93	20.8	49	52.7*
Easily fearful	92	20.6	59	64.1*
Easily gets tired	89	19.9	36	40.4**
Decreased socialisation	84	18.8	56	66.7*
Anxiety, worry	65	14.5	33	50.8*
Indecisiveness	60	13.4	26	43.3**
Difficulty in thinking	32	7.2	22	68.8*

*p < 0.001; **p < 0.05; ***p < 0.01

### Difference in high and low exposure areas

Prevalence of many associated symptoms also differed significantly in high and low exposure areas. Continuing fear that the cyclone may come again was more frequent in HEA (60.4%) vs. that in LEA (23.5%) (χ^2^: 59.1, p: 0.000). It was particularly important as most children reported fear and reliving of experiences with slight wind and rain. Depressive symptoms were also prominent; and the following significantly differentiated subjects in HEA and LEA: depressed mood (HEA 37.3% vs. LEA 14.5%, χ^2^: 27.5, p: 0.000), hopelessness (HEA 38.1% vs. LEA 10.1%, χ^2^: 42.8, p: 0.000), decreased interest in pleasurable activity (HEA 49.6% vs. LEA 5.0%, χ^2^: 98.4, p: 0.000) and decreased social interaction (HEA 28.4% vs. LEA 4.5%, χ^2^: 40.1, p: 0.000).

Binary logistic regression suggested that high exposure, lower educational level and middle socioeconomic status significantly predicted the outcome of PTSD. The summary is provided in table 4.

**Table 4:** Summary of binary logistic regression analysis for variables predicting PTSD

	B	S.E.	Wald	df	Sig.	Exp(B)	95.0% CI for Exp(B)	
							Lower	Upper

High exposure	1.411	.294	23.022	1	.000	4.100	2.304	7.296
Age	.251	.138	3.277	1	.070	1.285	.979	1.685
Education years	-.336	.126	7.102	1	.008	.714	.558	.915
Received disaster warning	.281	.229	1.496	1	.221	1.324	.844	2.076
Below poverty line SES#			8.503	2	.014			
Lower SES	.430	.291	2.187	1	.139	1.537	.869	2.716
Middle SES	.841	.296	8.066	1	.005	2.319	1.298	4.145
Constant	-2.875	1.067	7.258	1	.007	.056		

SES: Socioeconomic status; # Below poverty line is taken as the reference category in SES groups; CI: Confidence Interval.

## Discussion

It is known that children may not report their psychological reactions to the trauma unless they are specifically asked about aspects of trauma [29]; and that directly asking the child about PTSD symptoms as they relate to the stressor is always required [1]. In the index study symptoms of PTSD were evaluated clinically, supported by a checklist questionnaire based on ICD-10-DCR [28]. It was feasible to assess children in the post-disaster situation in schools and villages through the above method.

Construct of PTSD has been criticised as having limited cross-cultural validity [22]. Our epidemiological study evaluated the construct for validity in children and adolescents in Orissa, India through clinical interview method. The results suggest that post-traumatic symptoms and the syndrome in children and adolescents in this culture resembled those noted in western societies.

Our study has a few limitations. There was no standardised instrument used for diagnostic purposes; however clinical interview method probably helped evaluating the cross cultural validity of the concept. Other vulnerability factors like pre-existing mental and physical ill health, other pre-existing or concurrent life events independent and unrelated to super-cyclone, pre-disaster functioning, coping strategies, history of distress or disorder in parents and family were not studied. There was no measure of post-disaster functioning which could have reflected the severity of psychiatric morbidity.

### Prevalence of PTSD

It was observed that a considerable proportion of children and adolescents had PTSD symptoms. The proportion of subjects having PTSD syndrome according to ICD-10-DCR at one year was 30.6% with an additional 13.6% having a probability of sub-syndromal PTSD. Similar proportions have been reported elsewhere [8] and in another study on victims of index disaster [7]. However as the prevalence figures of PTSD vary considerably amongst studies it is probable that various factors contribute to the outcome. The relatively higher prevalence in the index study might be due to the greater severity and prolonged period of trauma. The disaster continued for three days, external support was not available for days and in some places for weeks in the high exposure areas.

### Prevalence of PTSD symptoms

A considerable proportion of children and adolescents reported post-traumatic symptoms. Experiencing distress when reminded or exposed to cues was the most common presentation. Visual cues to the disaster in the form of damaged environment and structures were everywhere even one year after the disaster, especially in HEA. It was inescapable from the cues of the trauma. This is particularly important as the enduring effects of disaster associated with 'traumatic reminders' are reported to be etiologically important for continuing psychological morbidity [9]. Continued exposure to cues and frequent interaction with the disaster workers triggering memories of the trauma may probably explain the observation that the proportion of children and adolescents reporting difficulty in recollecting were less in HEA compared to LEA. Though all children with PTSD had actual or preferred avoidance, the proportion of all children reporting this from HEA was less than that from LEA. Prevalence of all other symptoms of PTSD was significantly more in HEA than LEA.

### Other symptoms associated with PTSD

Depressive and anxiety symptoms were present in many children and adolescents with PTSD. The most common symptom was a continuing fear that the cyclone may come again. Fear of reoccurrence was the most frequent symptom one year after a flood elsewhere [5]. There was frequent panicky behaviour reported whenever there was high wind or rain. There were symptoms of anxiety, worry and becoming easily frightened. The presence of anxiety symptoms is comparable with post-disaster sequel studies elsewhere [8,11,30]. However we did not find disruptive behaviour, nor was it reported by the parents in the post-disaster scenario as reported elsewhere [8].

Depressive symptoms like depressed mood, anhedonia, feeling tired most of the time, hopelessness and indecisiveness were common and were significantly associated with PTSD diagnosis. Somatic symptoms like headache and decreased appetite were common as well. Almost one in four subjects had depression as a diagnosis and it was a comorbid condition in one third of PTSD cases; which was a significant association compared to the subjects without PTSD. Association of depression with PTSD in a post-disaster scenario has been reported frequently [7,8,21,31]. However, as most of the children and adolescents with PTSD did not have syndromal depression and vice versa; and one disorder could not predict the presence of other; it appears that they are probably distinct entities. The significant association, however, is probably due to an overlap of post-disaster psychiatric symptoms, which are known to be a conglomeration of PTSD, depression and anxiety symptoms [7].

### Parental report

Most of the children and adolescents who were reported to have post-disaster mental health problems by parents and teachers received a syndromal diagnosis. However in the majority of cases (87.2%) parents and teachers did not perceive the mental ill health in the children following disaster, similar to observations in other studies [1]. The observation that parents often do not 'see' the suffering in their children suggests the need for public education on post-disaster psychiatric sequel and active screening of the vulnerable population.

### Vulnerability factors

#### Gender

A greater percentage of female children were affected than males, however the difference was not statistically significant. It has been reported that female gender is more vulnerable in many studies [14,16,32,33]; however there are other studies who have failed to observe gender differences [5,34]. It is suggested that gender difference, if existent, may depend upon various factors, and may get obliterated by higher exposure to trauma [21].

#### Age

It is known that age at exposure to the traumatic event mediates the prevalence of PTSD [35,36]. Subjects with PTSD had lower mean age than those without the diagnosis. In the age groups, 11–13 year-olds were more vulnerable. After Hurricane Hugo preadolescents were reported to be more likely to have PTSD [2]. However in contrast, prevalence of PTSD in adolescents after Hurricane Andrew increased with age [4]. There is a suggestion that cognitive immaturity may protect the younger children from appreciating the implications of the disaster [19]. However, it could be possible that very young children may not be able to verbalize their feelings; or the manifestation may be different due to developmental stage. Differences in post-disaster psychological responses have been reported across a broad spectrum of developmental stages in children [37]. The lower prevalence in older adolescents may be due to factors like greater resilience or coping strategies compared to that of younger ones, however this issue was not specifically studied.

#### Education

Our study suggested that children up to class 5 educational level were more vulnerable than those in higher classes. Their mean age was 10.5 years which corresponds to the increased vulnerability of preadolescents. Similar findings have been reported following Hurricane Hugo [2]. Higher educational level may itself be protective, besides increased age; however this issue may need further study.

#### Socioeconomic status

There was a significant relationship between SES and prevalence of PTSD. It was highest in the middle SES followed by lower and least in those who were below poverty line. Similar finding has been reported in victims of super-cyclone from other areas [7]. It is understandable that the economic impact on the middle SES families was greatest amongst the groups; and adjustment after losing almost everything was appreciably difficult for this group [7,26]. The link between resource loss and stress making people vulnerable to psychological disorder is well established [38,39].

#### Disaster warning

Interestingly more children with PTSD reported having prior information about the super-cyclone. There is a report of existence of a mild but prevalent PTSD-like reaction from exposure to a prediction of disaster [40]. As trait anxiety has been found to be a risk for the development of severe post-traumatic reactions [41], role of state anxiety before disasters contributing to post-disaster symptomatology may be explored. It is worth studying the relation of disaster warnings, anticipatory stress reactions and vulnerability of development of PTSD. This may provide insights into the development of methods for providing support to children during disaster warnings.

#### Degree of exposure to disaster

Significantly more children and adolescents from HEA had PTSD. However as the disaster related damages, stay in shelters, death in family, and degree of fear during super-cyclone were comparable amongst children with or without PTSD; it suggested the probability of other factors predicting the outcome. Intensity of the exposure to the disaster and experience of post-trauma adversities could be some of them. In these accounts, children and adolescents in HEA had the worse experience. In addition to significantly more children reporting damage to home, displacement, stay in shelters, death in family, and extreme degree of fear compared to those in low exposure; there were considerable post-disaster adversities. Relief and external support could not reach them for days and in some places weeks after the super-cyclone. The victims in HEA suffered a prolonged period of helplessness with no contact with the outside world, starvation, no shelter and physical hardships. It is known that the degree of exposure, extent of loss of family members [13,15], accumulation of multiple stressors [16-18] and coexisting adverse circumstances [19] increase the vulnerability. In addition, PTSD symptoms are known to be significantly related to the proximity to the epicenter, exposure to threat [32] and severity of disaster [33]. These factors were more appreciable in children in HEA.

### Determinants of PTSD

Binary logistic analysis suggested that high exposure, lower educational level and middle SES were significant determinants of PTSD as an outcome following the super-cyclone. Higher exposure to the disaster has been well established to be predictive of PTSD in children and adolescents following disasters [6,32,33,41]. Though many studies suggested female gender being more vulnerable [2,6,16,33,41] and in our study more number of females were affected; gender could not significantly predict the outcome. Children and adolescents who had PTSD in our study had slightly but significantly lower age compared to those without, but it did not appear to be independently contributing to the outcome, unlike reports of many studies [2,33,41]. However lower educational level, with an obvious confounding factor of lower age, has been found to be a determinant of PTSD in regression analysis.

## Conclusion

The study clinically validated the presence of PTSD syndrome in children and adolescents one year after the super-cyclone in Orissa. A considerable proportion of children and adolescents exhibited post-traumatic symptoms, and almost one third could be diagnosed as having PTSD by ICD-10-DCR criteria. Parents and teachers could identify mental health problems only in a small proportion of children and adolescents with syndromal diagnosis. High exposure, lower educational level and middle socioeconomic status were significant determinants of PTSD. It appears that PTSD in children and adolescents can go unnoticed even following disasters and reach an epidemic proportion. Systematic clinical screening may provide the information required for the disaster mental health programme.

## Competing interests

The author(s) declare that they have no competing interests.

## Authors' contributions

NK conceptualized, wrote protocol, collected and analyzed data, wrote the manuscript. HK translated the protocol and questionnaires to Oriya, helped in the design and coordination; all authors were involved in the study in affected areas, collected data, read and approved the final manuscript.

## Pre-publication history

The pre-publication history for this paper can be accessed here:


